# The Children’s Somatic Symptoms Inventory-8: Psychometric Properties of a Brief Measure of Somatic Distress

**DOI:** 10.3390/children11111326

**Published:** 2024-10-30

**Authors:** Amanda L. Stone, Judy Garber, Lynn S. Walker

**Affiliations:** 1 Department of Anesthesiology, Vanderbilt University Medical Center, Nashville, TN 37232, USA; 2 Department of Pediatrics, Vanderbilt University Medical Center, Nashville, TN 37232, USA; 3 Department of Psychology and Human Development, Vanderbilt University, Nashville, TN 37235, USA

**Keywords:** chronic pain, functional neurological symptom disorder, somatic symptoms disorder, widespread pain, anxiety, depression

## Abstract

Background: Children often present to primary and specialty care clinics with multiple somatic symptoms of nonspecific origin that can be highly distressing and prompt significant health service use. We evaluated the psychometric properties of the eight-item Children’s Somatic Symptoms Inventory (CSSI-8) as a brief measure of somatic distress that could be easily integrated into clinical systems. Method: Eight items from the 24-item CSSI were selected based on their representation of multiple bodily systems, association with high base rates, and ability to maximize the separation of the items’ Rasch measure scores. The psychometric quality of the eight-item scale was evaluated in 876 pediatric patients with chronic abdominal pain and a nonclinical sample of 954 school children using methods from three psychometric models (the classical test theory, Rasch modeling, and confirmatory factor analysis). Results: The CSSI-8 showed good measurement properties on an extensive array of psychometric criteria, had adequate Rasch person separation reliability for a brief instrument (r_sep_ = 0.74–0.75), and distinguished between clinical and nonclinical youth. Girls in both groups had significantly higher CSSI-8 scores than boys. Norms for the clinical sample are presented. Conclusions: The CSSI-8 is a psychometrically sound measure suitable for use as a brief dimensional assessment of pediatric somatic distress.

## 1. Introduction

A substantial proportion of somatic symptoms presented by patients in pediatric primary care and subspecialty clinics are nonspecific; that is, they cannot be linked to tissue damage or pathology [1]. Nonetheless, these symptoms may be associated with high levels of distress, disability, and health service utilization [2,3] and may persist into adulthood [4,5]. Although a single symptom, such as abdominal pain, may be the primary focus of concern at the time of a clinic visit, individuals with one medically unexplained symptom often have additional somatic symptoms that cannot be linked to a recognized medical condition [6,7]. The experience of somatic symptoms can be placed on a continuum of severity that varies from underreporting to excessive reporting of symptoms [8]. Otherwise healthy individuals in the community, as well as clinical patients, may experience levels of somatic distress unrelated to the presence or severity of any underlying organic disease [9,10,11,12,13].

The Children’s Somatic Symptoms Inventory (CSSI-24), developed by Walker and colleagues [14], has been used in clinical and community settings for the in-depth assessment of pediatric somatic distress [15,16,17,18,19,20]. The CSSI-24 is a self-report measure of the severity of 24 somatic symptoms. The measure has demonstrated reliability and validity across cultures and in both clinical and community samples [7,15,21,22,23,24,25,26,27]. Additionally, the measure has predicted outcomes in longitudinal studies of youth posttraumatic stress symptoms [28], functional gastrointestinal disorders [6,29], and prolonged post-concussion symptoms [30]. Although the CSSI-24 is a good choice for research, an even shorter instrument may be more efficient for clinical assessment and could also be used to reduce participant burden in research.

In the current study, we developed an eight-item version of the Children’s Somatic Symptoms Inventory (CSSI-8) using three main approaches to psychometrics—the classical test theory [31,32], item response theory using Rasch modeling [33], and factor analysis. This multiple-model evaluation provided an in-depth psychometric assessment [27,34,35]. We aimed to develop a brief tool that had a high correlation with the CSSI-24 and that reliably differentiated between clinical and community samples of youth.

## 2. Materials and Methods

### 2.1. Participants and Procedure

This study was approved by the Vanderbilt University Institutional Review Board (protocol #990658, approved 21 February 2002). Parents gave consent and youth gave assent for participation.

#### 2.1.1. Patient Sample

CSSI-24 data for the current study were collected from several cohorts of consecutive new patients evaluated for unexplained chronic or recurrent abdominal pain in a tertiary care pediatric gastroenterology clinic (see [27] for details). Eligibility criteria included being between the ages of 8 and 18 years old, living with parent(s) or a parent figure, speaking English, having no chronic illness or preexisting organic disease that would explain the abdominal pain, and no developmental disability that would preclude study participation. The CSSI-24 was administered at the clinic prior to the child’s medical evaluation by an interviewer who read the items aloud and asked children to indicate their answers on a printed response sheet. The total patient sample included 876 youth (mean age = 11.66; SD = 2.47), 59% were female, and it was predominantly Caucasian (87.8%).

#### 2.1.2. Community Sample

The community sample (*n* = 954) comprised youth who completed the CSSI-24 as part of a health survey conducted in several public schools. Youths with a developmental disability that required placement outside the regular classroom were excluded. This sample had a mean age of 11.17 years (SD = 1.04), was 55% female, and predominantly Caucasian (94.3%).

### 2.2. Measures

Children’s Somatic Symptoms Inventory (CSSI-24), Self-Report Form [14,27]. Youth completed the Self-Report Form of the CSSI: a questionnaire that assesses the perceived severity of 24 somatic symptoms. The CSSI includes items from the symptom criteria for somatization disorder as defined by the DSM-III-R [36] and from the somatization factor of the Hopkins Symptom Checklist (HSCL, [37]). The stem for the CSSI-24 is the same as that in the HSCL, namely, “How much were you bothered by each (symptom)?” The standard time period for symptom reporting on the CSSI-24 is 2 weeks. The 5-point response scale ranges from “not at all” (0) to “a whole lot” (4). Total CSSI-24 scores are calculated by summing all item ratings.

### 2.3. Item Reduction

The 876 participants in the clinical sample were split into two groups by a SAS-generated random number to create a “learning” sample (*n* = 417) and a cross-validation sample (*n* = 459). This split allowed us to develop the CSSI-8 with the learning sample and then examine its psychometric properties with fresh cases in the cross-validation patient sample and the community sample (*n* = 954).

Psychometric properties of the CSSI-24 items have been presented elsewhere [27]. Items on the CSSI-24 were reviewed and selected for a current, more concise measure to (1) represent multiple bodily systems, (2) be associated with excessive symptom reporting, as noted in the literature, and (3) maximize the separation of the items’ Rasch measure scores.

Table 1 shows the 8 items selected for the short form and compares these items to those included in two other widely used measures of somatic symptoms: the somatoform disorders scale of the original version of the Patient Health Questionnaire (PHQ) for adults [38] and the somatic complaints subscale of the Child Behavior Checklist (CBCL) [39]. Seven of the eight items selected for the CSSI-8 are based on one or both instruments, suggesting that the CSSI-8 comprises items commonly assessed using measures of somatic symptoms that are widely used in pediatric and adult populations. The eighth item, “weakness,” which does not appear on the PHQ or the CBCL, was included because it reflects general malaise and, therefore, represents more bodily systems than other CSSI-24 items with similar measure scores.

### 2.4. Statistical Methods for Evaluating the CSSI-8

Next, we evaluated the CSSI-8 in the learning sample using criteria taken from the classical test theory, confirmatory factor analysis, and Rasch modeling. A limitation of the classical test theory and factor analysis is their complete reliance on correlational methods to assess the psychometric properties of tests. In contrast, the Rasch model [33,40] offers a more rigorous logistic measurement model as a one-parameter member of the item response theory (IRT) family [41]. The IRT measurement model evaluates persons and test items in common units on a single scale. Originally, Rasch models [40] focused on dichotomous (correct vs. incorrect) test items that used measures of academic performance, but in recent decades, Rasch models have been applied to a wide range of topics. In the current study, Rasch estimation was performed with Winsteps V3.58 [42] using a rating scale model for ordered response categories 0, 1, 2, 3, and 4 [40].

To evaluate the CSSI-8 short form, we examined four broad criteria and devised “warning flags” that focused on possible problems. Because the flags were arbitrary, they should not be considered “pass-fail” rules; rather, the flags call attention to possible problems with items or the whole test.

1. Descriptive statistics (mean, skew, and kurtosis) warn of floor or ceiling problems that reduce an item’s variance. If a test item is nearly constant (the same for everyone), its contribution to the total score may be weak. Floor/ceiling problems also may occur when an item’s mode reaches the item’s minimum or maximum or when items have skew or kurtosis >3.
2. Corrected item-total correlations (r_it_) show items that contribute to Cronbach’s alpha internal consistency reliability. Items with r_it_ <0.4 may contribute less.
3. A single-factor confirmatory factor analysis (CFA) evaluates the test’s “factorial validity” for a one-factor measurement model. Warning flags highlight low-load items with standardized betas <0.4; such items may not measure the test’s main factor well. The measurement model’s fit to the data can be evaluated with a deviance-based fit index and a residual-based fit index. For example, using Bentler’s Confirmatory Fit Index (CFI; [43]), values “close to” 0.95 are considered adequate [44]. Using a residual-based fit measure such as the SRMR (Standardized Root Mean Squared Residual), values “close to” 0.08 are considered adequate [44]. Poor fit indices raise questions about the single-factor measurement model’s fit to the data.
4. Infit, outfit, and person separation reliability evaluate a test’s fit to a Rasch measurement model. Infit measures the model’s fit for closely targeted items, and outfit measures the fit for far-targeted items. Items with an infit or outfit outside the interval 0.5–1.5 may be problematic [45]. Even stricter limits for infit and outfit have been suggested [46]. Person separation reliability assesses a test’s ability to measure differences among people. For most tests, reliability should be > 0.80, although for a short tool such as the CSSI-8, a reliability of > 0.70 is acceptable [47].

## 3. Results

Table 2 shows the item statistics for the eight CSSI-8 items in the learning sample, with certain items flagged with asterisks for review. Of the 72 cells in Table 2, 5 cells are flagged. Three of the flagged cells refer to a single item, “heart beating too fast.” These flags (low mean, high kurtosis, low item-total r) may be a result of floor problems, with very few children endorsing heartbeat problems. Nonetheless, the Rasch criteria (infit and outfit) and the factor loadings were good, suggesting that the item works. “Heart beating too fast” was retained to maximize the range of measure scores and because it is the only item on the CSSI-8 that represents the cardiac system. Overall, Table 2 shows some minor concerns, but all eight items in the CSSI-8 seem to be adequate. Although three items have item-total correlations <0.40, all are 0.35 or higher, and infit–outfit estimates indicate that all items fit the Rasch model.

After evaluating the items for the clinical learning sample in Table 2, we reviewed whole-test statistics to evaluate the psychometric properties of the CSSI-8 in both the learning sample (*n* = 417) and the cross-validation sample (*n* = 459), as shown in Table 3. Psychometric performance in the two samples was very similar. Both reliability estimates (i.e., Cronbach’s alpha and Rasch person separation reliability) were adequate for a short form (0.74 to 0.75). Only two warning flags occurred, both for Bentler’s CFI, indicating a poor fit to a single-factor confirmatory model (good CFI = 0.95 or higher). This lack of fit to a unidimensional model was consistent with our earlier findings with the CSI-24 [27]. Thus, like the CSSI-24, the CSSI-8 short form is composed of items all with a general factor in common (i.e., somatic symptom reporting), though each item has something unique (viz., different bodily systems). Although the CSSI-8 is not perfectly unidimensional, adding its items into a total score makes psychometric sense given the adequate alpha reliability and fit of the Rasch model, as well as clinical sense given the need for a single measure capturing overall somatic distress regardless of the bodily system.

### 3.1. Item Targeting in the CSSI-8

“Targeting” refers to the match between measure scores for an item and a given child. The Rasch model measures both children and items on the same scale so we can examine the targeting of items for the children in the sample. Rasch and IRT models help test designers invest items where they are most needed to accomplish the test’s purpose.

**Figure 1:**
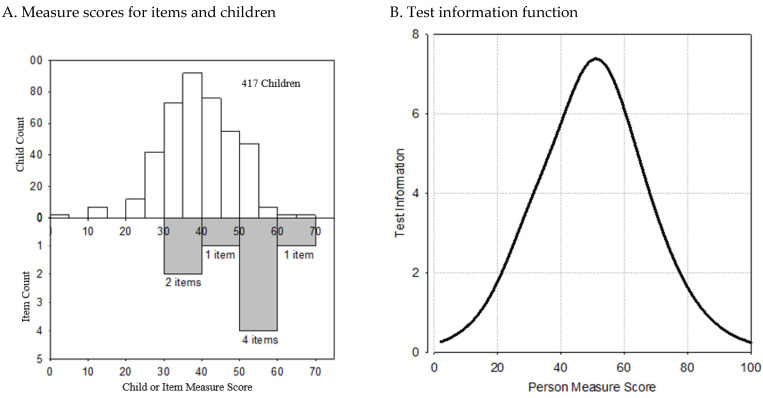
Item targeting for the CSSI-8 in the learning sample. (**A**) Items and children with CAP by total score. Most of the items are targeted toward above-average children. (**B**) CSSI-8 measures most precisely for children with total scores of 40–60.

Figure 1A shows the distributions of measure scores for 417 youth in the clinical sample and, in gray, for the eight CSSI-8 items. When an item and a child have similar measure scores (“close targeting”), there is more information about the child (smaller standard error). In Figure 1, five of the eight CSSI-8 items had measure scores from 40 to 60. Comparing these item scores to youth measure scores suggests that the CSSI-8 items match youth with above-average measure scores.

Figure 1B shows the whole-test information function for the CSSI-8. As expected, the most precise information occurs for children with measure scores in the 40–60 range, suggesting that the CSSI-8 is most accurate for clinical children in the 55th to 99th percentile, namely those who are above the median of the clinical sample. Children with measure scores below 20 can be measured, but their scores will have large standard errors of estimate.

### 3.2. Replication in a Community Sample

In the community sample (*n* = 954), the CSSI-8′s Cronbach’s alpha was 0.80, the Rasch person separation reliability was r_sep_ = 0.70, and item-total correlations were 0.42–0.64. Infits (0.72–1.47) and outfits (0.69–1.34) were satisfactory. These results suggest that the CSSI-8 measured the nonclinical sample adequately.

### 3.3. Relation of CSSI-8 Total Scores to Sample Type and Sex

CSSI-8 total scores for the clinical sample (M = 9.68, SD = 5.12) were significantly higher than for the nonreferred community sample (M = 7.96, SD = 5.82), Wilcoxon *p* < 0.0001, with an effect size of d = 0.31 (Cohen classified small/medium/large effects as d = 0.2/0.5/0.8) [48]. In the combined clinical and community sample, CSSI-8 total scores were significantly higher for girls (M = 9.49, SD = 5.73) than boys (M = 7.82, SD = 5.20), Wilcoxon *p* < 0.0001, with an effect size of d = 0.30. A two-way analysis of variance revealed that the group (clinical vs. community) divided by sex interaction was not significant, F(1, 1855) = 0.14, *p* = 0.71, suggesting that the sex difference in CSSI-8 total scores was similar in the clinical patient and nonreferred community samples.

### 3.4. Relation of the CSSI-8 to the CSI-24

Finally, we calculated the correlations between the CSSI-8 and CSI-24. The CSSI-8 was highly correlated with the CSI-24 in both clinical samples (r = 0.90 in the learning sample; r = 0.91 in the cross-validation sample) and the community sample (r = 0.94). These correlations suggest that the CSSI-8 and the CSI-24 are measures of the same construct.

### 3.5. Scoring the CSSI-8

Administration and scoring instructions for the CSSI-8 are presented in Supplementary Materials File S1. If equal-interval scores are preferred, the Rasch measure scores may be used rather than the CSSI-8 total sum scores. Rasch measure scores corresponding to each CSSI-8 total score appear in a lookup table in Supplementary Materials File S1. Percentile scores are also provided and may enable clinicians to compare their patients to those in the *n* = 876 clinic sample from this study.

## 4. Discussion

Somatic distress is common in children and adolescents and is relevant, particularly for youth presenting with functional impairment across multiple life domains (e.g., school, activities, sleep) and/or frequent health service use. We used learning (*n* = 417) and cross-validation (*n* = 459) clinical samples of pediatric patients with chronic abdominal pain and a nonclinical school sample (*n* = 954) to develop a brief dimensional measure of pediatric somatic symptom distress. The CSSI-8 had adequate reliability for a short form (r_sep_ = 0.74–0.75), was highly correlated with the CSSI-24 (r = 0.90–0.94), and showed good measurement properties on an extensive array of psychometric criteria.

Notably, the items chosen for inclusion in the CSSI-8 represent symptoms frequently observed to co-occur in youth presenting with chronic overlapping pain conditions [49]. The proposed mechanisms underlying the overlap between chronic abdominal pain and other functional pain syndromes include physiological processes (e.g., dysregulations in immunologic and neuroendocrine function, central sensitization of pain) [50], cognitive–affective factors (e.g., enhanced threat response, hypervigilance towards symptoms) [51], and intergenerational processes (e.g., social learning, genetic predisposition) [52,53,54,55].

The CSSI-8 was designed to capture the affective experience of symptoms with the item stem—“In the past 2 weeks, how much were you *bothered* by each symptom?” Thus, the CSSI-8 focuses on the interpretation of symptoms as problematic or bothersome to the individual more so than the frequency of symptoms. Although, historically, a high burden of ambiguous somatic symptoms was considered a marker of a psychiatric disorder [56], it is important to note that there is a new movement toward normal psychology of somatic sensations seeking to understand the ways individuals experience and interpret sensations, not as a sign of pathology, but as a process that shapes development and behavior [57].

Similarly, clinical professionals in recent years have cautioned against over-pathologizing youth who present with multiple “medically unexplained” somatic complaints as it could lead to greater stigma, reduced therapeutic alliance, and inappropriate treatment [54,58]. On the other hand, providing a psychiatric diagnosis such as a somatic symptoms disorder when appropriate could positively guide treatment by helping to reduce medicalization and healthcare seeking when it is unlikely to lead to symptom relief [58]. The CSSI-8 may be a useful tool for assessing the severity of somatic distress and help guide diagnostic assessment and treatment planning/response when combined with other clinical information (e.g., functional impairment due to symptom-related distress, medical assessment of symptom severity, time and energy devoted to symptom concerns).

The strengths of the present study include the use of IRT to evaluate the properties of the items and develop the short form, the ability to test item performance across both clinical and nonclinical samples of youth, and the clinical utility of an eight-item short form. Primary limitations include the predominantly Caucasian population and the evaluation of the measure in only one clinical population (i.e., abdominal pain). The extent to which the psychometric properties of the CSSI-8 generalize to more diverse or heterogeneous clinical populations is unknown.

The CSSI-8 is a brief and efficient tool to assess somatic symptom reporting in school-aged children and adolescents. Because the CSSI-8 is so brief, it places low demands on respondents and can be completed easily in a clinic waiting room. Additionally, the CSSI-8 can be administered with minimal training and is easily scored by summing the endorsed items. Interpretation is facilitated by norms that allow clinicians to compare their patients’ scores to percentile ranks for this study’s large sample of youth with chronic abdominal pain, one of the most common somatic complaints of childhood.

Like the CSSI-24, the CSSI-8 discriminates well between clinical and nonreferred youth. Consistent with the literature on pediatric somatic symptoms [2,59,60], CSSI-8 scores were significantly higher in girls than boys in both the patient and community samples. Further study is needed to establish the validity of the CSSI-8 in patients across diverse clinical settings and in ethnically diverse populations. Finally, studies are needed to assess the acceptability and utility of the CSSI-8 in clinical settings, as well as its utility in identifying youth at risk of poor health outcomes and high health service use.

## Figures and Tables

**Table 1:** Somatic Symptoms on the CSSI-8, PHQ, and CBCL**.**

			Measures	
Items				
		CSSI-8	PHQ	CBCL
Stomach Pain		X	X	X
Headache		X	X	X
Back Pain		X	X	X^1^
Faintness or Dizziness		X	X	
Arm or Leg Pain		X	X	X^1^
Heart Beating Fast		X	X	
Nausea		X	X	X
Weakness		X		
Constipation			X	
Shortness of Breath			X	
Menstrual Cramps			X	
Pain w/Intercourse			X	
Problems w/Eyes				X
Rashes				X
Vomiting				X

^1^ Represented by one item on the CBCL (“aches and pains”). Note. CSSI-8 = Children’s Somatic Symptoms Inventory; PHQ = Patient Health Questionnaire; CBCL = Child Behavior Checklist.

**Table 2:** Statistics for evaluation of the final 8 items on the CSSI-8 (*n* = 417).

Variable	Mean	Std Dev	Skew	Kurtosis	Item-Total r	Std CFA Beta	Measure	Infit	Outfit
Stomach Pain	2.56	1.15	−0.42	−0.68	0.44	0.53	33.33	0.90	0.98
Headache	1.35	1.18	0.48	−0.69	0.43	0.50	46.89	1.01	1.00
Weakness	0.87	0.98	1.02	0.46	0.53	0.64	53.07	0.78	0.73
Lower Back Pain	0.77	1.10	1.28	0.58	0.38 *	0.44	54.64	1.30	1.20
Faintness	0.68	0.98	1.44	1.43	0.47	0.56	56.14	0.99	0.92
Arm or Leg Pain	0.59	0.92	1.54	1.72	0.35 *	0.41	57.80	1.21	1.08
Heart Too Fast	0.42 *	0.90	2.28	4.51 *	0.38 *	0.47	61.40	1.36	1.33
Nausea	2.27	1.20	−0.21	−0.86	0.45	0.54	36.72	0.93	0.96

* indicates values as follows: mean < 0.5, skew > 3, kurtosis > 3, Item total correlation < 0.40, standardized beta from CFA < 0.40; infit and outfit, outside 0.5–1.5 [45].

**Table 3:** CSSI-8 performance in learning (*n* = 417) and cross-validation (*n* = 459) clinical samples.

Sample	Mean (Sum)	Std Dev	Skew	Kurtosis	Cron. Alpha	SRMR	Bentler CFI	Person Sep Rel
Learning N = 417	9.51	5.00	0.59	0.04	0.74	0.07	0.82 *	0.75
Cross-validation N = 459	9.84	5.22	0.64	−0.02	0.74	0.06	0.85 *	0.74

* indicates value outside of recommended range. Recommended ranges are as follows: Cronbach’s alpha internal consistency reliability > 0.70, SRMR (Standardized Root Mean Square Residual) < 0.08, Bentler’s CFI (Comparative Fit Index) ≥ 0.95, and Rasch person separation reliability > 0.70.

## Data Availability

The datasets presented in this article are not readily available because they are a part of a large dataset with privacy restrictions. Requests to access the datasets should be directed to amanda.l.stone@vumc.org.

## Supplementary Materials

The following supporting information can be downloaded at https://www.mdpi.com/article/10.3390/children11111326/s1, File S1: CSSI-8 Scoring Information
