# Supplementary material for: The Children’s Somatic Symptoms Inventory-8: Psychometric Properties of a Brief Measure of Somatic Distress
Source: Children (Basel). 2024 Oct 30;11(11):1326. doi: 10.3390/children11111326 (PMC11593210; doi:10.3390/children11111326)
Supplement: Supplementary file 1 [file children-11-01326-s001.zip › children-3231638-supplymentary.pdf]

CSSI-8 (Child Report)

Your Symptoms

Below is a list of symptoms that children and teenagers sometimes have. Circle a number telling how much you were bothered by each symptom during the past two weeks.

In the last 2 weeks, how much were you bothered by each symptom?

	Not at all	A little	Some	A lot	A whole lot
1. Pain in your stomach or abdomen (stomach aches)	0	1	2	3	4
2. Headaches	0	1	2	3	4
3. Pains in your lower back	0	1	2	3	4
4. Faintness or dizziness (feeling faint or dizzy)	0	1	2	3	4
5. Pain in your arms or legs	0	1	2	3	4
6. Your heart beating too fast (even when you're not exercising)	0	1	2	3	4
7. Nausea or upset stomach (feeling like you might throw up, or having an upset stomach)	0	1	2	3	4
8. Weakness (feeling weak) in parts of your body	0	1	2	3	4

CSSI-8 (Parent Report)

Your Child's Symptoms

Below is a list of symptoms that children and teenagers sometimes have. Read each one and circle a number telling how much your child was bothered by each symptom during the past two weeks.

In the last 2 weeks, how much was your child bothered by each symptom?

	Not at all	A little	Some	A lot	A whole lot
1. Pain in stomach or abdomen (stomach aches)	0	1	2	3	4
2. Headaches	0	1	2	3	4
3. Pains in the lower back	0	1	2	3	4
4. Faintness or dizziness	0	1	2	3	4
5. Pain in arms or legs	0	1	2	3	4
6. Heart beating too fast (even when not exercising)	0	1	2	3	4
7. Nausea or upset stomach	0	1	2	3	4
8. Weakness in parts of the body	0	1	2	3	4

CSSI-8

Item Sum Score	Rasch Measure Score	Percentile
0	4	1
1	16	1
2	23	4
3	27	7
4	30	12
5	32	19
6	34	27
7	37	35
8	38	43
9	40	50
10	42	57
11	43	64
12	45	70
13	46	75
14	48	80
15	49	84
16	50	88
17	52	91
18	53	93
19	54	95
20	55	96
21	57	97
22	58	98
23	60	99
24	61	99
25	63	99
26	65	99
27	67	99
28	70	99
29	73	99
30	77	99
31	85	99
32	97	100

Calculating a Total Score for the CSSI-8

A. If the CSSI-8 has no missing items, compute the total score as the sum of the 8 items. Items are scored 0–4. For the CSSI-8, item sum scores can range from 0 to 32.

B. If the CSSI-8 has missing items, compute the total score in two steps:

   1. Calculate the average item score from all nonmissing items.

   2. Multiply the average item score by 8.

C. If there are fewer than 6 completed items, researchers may treat the CSSI-8 as missing or incomplete.

D. Researchers who want equal-interval scores may look up the Rasch measure score that corresponds to the Item Sum.
